# Integrating Health Behavior Theories to Predict COVID-19 Vaccine Acceptance: Differences between Medical Students and Nursing Students

**DOI:** 10.3390/vaccines9070783

**Published:** 2021-07-13

**Authors:** Hila Rosental, Liora Shmueli

**Affiliations:** Department of Management, Bar-Ilan University, Ramat Gan 52900, Israel; hila.rosental@mail.huji.ac.il

**Keywords:** SARS Coronavirus, Health Belief Model, healthcare workers, Theory of Planned Behavior, vaccine acceptance

## Abstract

Background: This study aimed to explore behavioral-related factors predicting the intention of getting a COVID-19 vaccine among medical and nursing students using an integrative model combining the Health Belief Model (HBM) and the Theory of Planned Behavior (TPB). Methods: A cross-sectional online survey was conducted among medical and nursing students aged > 18 years in their clinical years in Israel between 27 August and 28 September 2020. Hierarchical logistic regression was used to predict acceptance of a COVID-19 vaccine. Results: A total number of 628 participants completed the survey. Medical students expressed higher intentions of getting vaccinated against COVID-19 than nursing students (88.1% vs. 76.2%, *p* < 0.01). The integrated model based on HBM and TPB was able to explain 66% of the variance (adjusted R^2^ = 0.66). Participants were more likely to be willing to get vaccinated if they reported higher levels of perceived susceptibility, benefits, barriers, cues to action, attitude, self-efficacy and anticipated regret. Two interaction effects revealed that male nurses had a higher intention of getting vaccinated than did female nurses and that susceptibility is a predictor of the intention of getting vaccinated only among nurses. Conclusions: This study demonstrates that both models considered (i.e., HBM and TPB) are important for predicting the intention of getting a COVID-19 vaccine among medical and nursing students, and can help better guide intervention programs, based on components from both models. Our findings also highlight the importance of paying attention to a targeted group of female nurses, who expressed low vaccine acceptance.

## 1. Introduction

On September 2020, more than 50 candidate vaccines were in the clinical evaluation stage, and no vaccine to COVID-19 was yet available. However, sooner than expected, in December 2020, the U.S. Food and Drug Administration (FDA) issued the first two emergency use authorizations (EUA) for COVID-19 vaccines. Such emergency authorizations were granted to Pfizer-BioNTech and Moderna for their vaccines designed to prevent COVID-19 [1]. Immediately after, Israel launched a national COVID-19 vaccination campaign, which has led to Israel having one of the highest rates of vaccinated individuals, with more than 49% of people having received at least the first dose by the end of February 2021 [2]. Nevertheless, despite vaccine availability, a significant part of the population will still not get vaccinated, partly due to a phenomenon known as vaccine hesitancy [3].

Surprisingly, vaccine hesitancy is present even among healthcare workers (HCWs), despite the great importance of vaccinating them. In fact, the CDC recommends HCWs be among the first to get a COVID-19 vaccine [4]. This prioritization has several reasons. First, HCWs are at an elevated risk of being exposed to COVID-19, as compared with the general public. Indeed, almost up to 50% of COVID-19 infections occur among HCWs [5]. Vaccinating HCWs can therefore help not only in preventing them from being infected, but also from further infecting others, and specifically vulnerable patients. Second, vaccinating HCWs can ensure adequate workforce and protect healthcare capacity. Lastly, At the policy level, HCWs play a key role in providing vaccine recommendations and counseling vaccine-hesitant patients [6,7,8]. A few recent studies that examined the intentions of HCWs to get vaccinated once a COVID-19 vaccine becomes available found that only 40–78% of HCWs were willing to get vaccinated, with doctors presenting higher rates than nurses [9,10,11,12,13,14].

Vaccinating medical and nursing students in their clinical years is also of high importance. These students are often found on the frontline in the battle against COVID-19, providing care for patients in COVID-19 departments, taking COVID-19 tests, and providing COVID-19 vaccines to patients. In addition, they play a key role in providing vaccine recommendations and counseling vaccine-hesitant patients as future professionals. Even fewer studies focused on this group of students [14,15,16], exploring their acceptance towards the novel COVID-19 vaccine. While these studies show that this group of students expresses high intention of getting vaccinated (77–98%), none of these studies made a clear distinction nor a comparison between medical students and nursing students.

In considering the factors associated with willingness to get vaccinated against COVID-19 among HCWs, demographic and health-related predictors were investigated in several studies. These studies found that significantly higher proportions of males [9,10,11,13], older subjects (above 60 years of age) [13] or who suffer from chronic illness [9], encounter COVID-19 patients [9] or work in COVID-19 departments and being a doctor [11] were willing to get vaccinated.

While only few studies have investigated predictors of COVID-19 vaccine acceptance, there are numerous studies reviewing predictors associated with acceptance of influenza vaccine among HCWs. In the present study, we adopted some of these related factors in addressing the current COVID-19 pandemic. Specifically, the literature reports several additional characteristics that describe HCWs who intended to get an influenza vaccine. These included religious subjects [17], those in a steady relationship or married [17,18], living with dependent children (under 21 years of age), have frequent contact with the elderly [17], or have family members with chronic illness [17]. Additionally, vaccine intentions could be predicted for HCWs who had higher income [19], worked at hospitals [20], especially in pulmonology departments [21].

Factors based on theoretical behavior models can also be valuable in predicting the willingness to get vaccinated. Theoretical models of health beliefs and risk perception are essential tools for understanding factors motivating and inhibiting health behavior. The Health Belief Model (HBM) [22] and the Theory of Planned Behavior (TPB) [23] are two of the most influential theories used to predict health behaviors. While HBM and TPB have been widely used for predicting behavior related to vaccination, to the best of our knowledge, they were not used in the context of COVID-19 vaccination among HCWs.

According to HBM, the intention of getting an influenza vaccine among HCWs depends on a number of factors, including: (1) perceived severity, namely, the perception of the seriousness and consequences following catching influenza for the individual and for others (e.g., loss of work time, pain and discomfort or financial); (2) perceived susceptibility, namely, risk perception of the likelihood of infection with influenza; (3) perceived benefits, namely the potential advantages of getting vaccinated against the virus (e.g., preventing the disease); (4) perceived barriers are the perceived obstacles relevant to vaccination (these can be physical such as side effects, psychological or financial); (5) cues to action, namely, factors that encourage a person to get vaccinated (internal factors, such as having experienced symptoms, or external factors, such as interactions with other people, information from the media or a physician’s recommendation) and (6) general health motivation [17,18,19,24,25,26,27].

According to TPB, the intention of getting an influenza vaccine among HCWs depends on a number of predictors, including [22,27,28]: (1) attitude towards vaccination; (2) subjective norms, which are the social pressures that people perceive from important others encouraging them to perform the behavior; (3) Perceived Behavioral Control (PBC), which is the degree of control a person believes he has over performing the behavior (i.e., the perceived ease or difficulty in performing the behavior) [23]; and (4) anticipated regret, which is the prospective feeling of positive or negative emotions after performing or not performing the behavior [28].

It is important to emphasize that HBM and TPB were also studied in the context of COVID-19 vaccination [29,30], but all of these studies examined the intentions of the general public and did not focus on HCWs.

The aim of this research was, therefore, to explore behavioral-related factors based on both HBM and TPB, in addition to demographic and health-related factors, for predicting the intention of getting a COVID-19 vaccine among medical and nursing students.

## 2. Methods

### 2.1. Study Design and Participants

We conducted a cross-sectional online survey among nursing and medical students in their clinical years in Israel. The survey was conducted between 27 August and 28 September 2020, before the second quarantine in Israel was announced.

Participants were recruited via opportunity sampling with a minimum overall target of 300 students per profession (nursing students, medical students). Inclusion criterion were being nursing or medical students in their clinical years, and 18 years of age and older.

At the beginning of the questionnaire form (see below), the respondents were informed that their participation was voluntary, they were permitted to terminate their participation at any time and that they confirmed informed consent to participate in the research. The interviewers followed a pre-defined closed-end protocol. Participants who refused to give their consent to proceed with the questionnaire and those under the age of 18 years were excluded. The questionnaire was in Hebrew.

### 2.2. Ethical Considerations

This study was approved by the Ethics Committee for Non-clinical Studies of Bar Ilan-University.

### 2.3. Questionnaire

**Figure 1:**
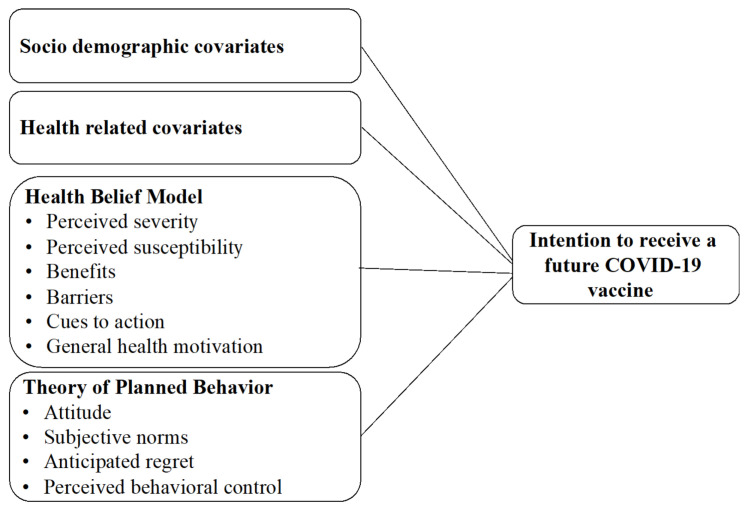
Conceptual Framework for the hypothesized predictors of intention to receive a COVID-19 vaccine.

The following sections describe the dependent and independent variables in the questionnaire and their operationalization in this study. The parameters comprising the study measurements used to build the conceptual model are described in Figure 1. The questionnaire consisted of the following sections: (1) HBM covariates; (2) TPB covariates; (3) intention to receive a future COVID-19 vaccine; (4) intention to receive an influenza vaccine; (5) concerns related to the COVID-19 vaccine; (6) sociodemographic covariates; and (7) health-related covariates.

Overall, the questionnaire consisted of 55 questions and took less than 10 min to complete.

### 2.4. Measurement and Variables

The dependent variable was the intention to receive a future COVID-19 vaccine, as measured by a one-item question on a 1–6 scale (1-strongly disagree to 6-strongly agree). The independent variables were: (1) Sociodemographic covariates, namely, age, gender, personal status, ethnicity, and socioeconomic level (based on the Israeli Central Bureau of Statistics scale and periphery level, defined by residential area); (2) health-related covariates, such as previous influenza infection, having received an influenza vaccine in the past 12 months (i.e., past behavior), suffering from a chronic disease, living with a person who belongs to a high-risk group, smoking, previous or current infection with the COVID-19 virus, having contact with COVID-19 patients (at hospital, at-home care or taking samples); (3) HBM covariates: Perceived susceptibility (included two items), perceived severity (included four items), benefits (included five items), barriers (included four items), cues to action (included five items), and general health motivation (one item); and (4) TPB covariates: Attitude (included two items), subjective norms (included two items), PBC (included four items), and anticipated regret (included one item). Items in the HBM and TPB models were measured on a 1–6 scale (1-strongly disagree to 6-strongly agree). Negative items were reverse scored, so that higher scores indicated higher levels of the item. Scores for each item were averaged to obtain each of the HBM and TPB independent categories.

### 2.5. Statistical Analyses

Data processing and analysis were done using SPSS for Windows (Version 25) software and the Process add-on for SPSS (Version 3.5) [31].

A Cronbach’s α internal reliability method revealed that the internal consistency of HBM was Cronbach’s α = 0.78 and that of TPB was Cronbach’s α = 0.80. The internal consistency of the integrated model was Cronbach’s α = 0.85. When divided into type of profession, the internal consistency of the model for nursing students was Cronbach’s α = 0.87 and Cronbach’s α = 0.82 for medical students.

To describe differences in variables between medical and nursing students, we conducted a series of chi-squared tests (for categorical variables) and *t*-tests (for numeric variables).

Next, we performed a univariate analysis to identify potential predictors and meaningful interactions among the sociodemographic and health-related variables. Specifically, we performed a series of two-way analyses of variance (ANOVA) tests. For each test, a single sociodemographic or health-related variable was taken, together with the profession variable, as the independent variables, and the intention of getting a COVID-19 test was taken as the dependent variable.

Finally, we performed a multivariate analysis to investigate determinants of intention to receive a COVID-19 vaccine. For this purpose, we performed a hierarchical logistic regression. The intention to receive a COVID-19 vaccine was used as the dependent variable measured by a one-item question on a 1–6 scale (1-strongly disagree to 6-strongly agree). The independent variables were divided into seven blocks. To avoid over-complexity and possible multi-collinearity, we used the stepwise procedure from the sixth step onwards. The significance level for all analyses was set to 0.05.

## 3. Results

### 3.1. Participant Characteristics

Overall, 628 respondents completed the online survey. Of these, 51% (*n* = 321) were medical students and 49% (*n* = 307) were nursing students. The average age of the medical students included in the sample was 28.06 years (SD = 3.33), while that of nursing students was 26.04 years (SD = 3.74). Among the categorical sociodemographic variables (see Table 1), significant differences were found between medical and nursing students in all cases, except for the having children variable. For example, among medical students, half of the respondents were female (*n* = 161) whereas among nursing students, the representation of females was significantly higher (*n* = 257, 83.7%). Among the health-related variables, significant differences were found for living with someone in risk, being exposed to COVID-19 patients at work, and having received an influenza vaccine in the previous season. For example, medical students reported having received an influenza vaccine in the previous season at a significantly higher level, as compared to nursing students (81.6% vs. 47.6%, *p* < 0.001). For completeness, Table S1 shows a comparison between medical and nursing students for each variable of the HBM and TPB models.

### 3.2. Intention to Receive Future COVID-19 Vaccine

Medical students expressed higher intentions of getting vaccinated against COVID-19 than did nursing students (88.1% vs. 76.2%, *p* < 0.01).

### 3.3. Univariate Analysis

Table 2 presents the significant main effects and interactions obtained when applying the two-way ANOVA tests. Recall that for each such test, the profession variable together with one of the sociodemographic or health-related variables were taken as the independent variables, and the intention of getting a COVID-19 vaccine was taken as the dependent variable. More specifically, only three main effects: socioeconomic status, COVID-19 infection and previous influenza vaccination, and a single two-way interaction, profession*gender, were found significant. All other variables were not found to be significant and hence were excluded from the table. Note that although gender did not present a significant main effect, since it did present a significant two-way interaction, we kept its main effect in Table 2, as well as in the multivariate analysis that we describe below.

### 3.4. Multivariate Analysis

Our integrated model included HBM and TPB variables, as well as sociodemographic and health-related variables, joined by hierarchical logistic regression so as to predict intention to receive a COVID-19 vaccine. The hierarchical logistic regression coefficients and process are presented in Table 3.

The final integrated regression model explained 66% of the variance in intention to receive a COVID-19 vaccine (adjusted *R* square = 0.66). In the first step alone, the profession variable was inserted as a predictor. In the second step, the significant sociodemographic variables (gender and socioeconomic status) were inserted as predictors. In the third step, the significant health-related variables (COVID-19 infection and previous vaccination against influenza) were inserted as predictors. In the fourth step, all HBM variables were inserted as predictors. In the fifth step, all TPB variables were inserted as predictors. In the sixth step, we inserted all two-way interactions between the profession variable and all of the HBM and TPB variables as well as the gender variable. However, to avoid over-complexity and possible multi-collinearity, we used the stepwise procedure from the sixth step onwards. Thus, in the sixth step, only the two-way interaction between profession and gender was inserted. Lastly, in the seventh step, only the two-way interaction between profession and susceptibility was inserted. All other two-way interactions were excluded from the model via the stepwise procedure. Importantly, as accepted, we centered all variables that define the interaction products and calculated the interactions between them prior to inserting them into the regression model [32].

From HBM, perceived susceptibility, benefits, and cues to action, and from TPB, attitude, self-efficacy and anticipated regret, were all positively significant predictors of intention of getting a COVID-19 vaccine. At the same time, barriers were a significant negative predictor of intention of getting a COVID-19 vaccine. Lastly, the two interaction terms (i.e., profession × gender and profession × susceptibility) were both found to also be predictors of intention of getting a COVID-19 vaccine. When broken into simple slopes, the interaction between gender and profession revealed the following: Males had a higher intention of getting vaccinated, relative to female only among nursing students (b=−0.29,se=0.15,p=0.04, 95% CI [−0.58,−0.001]). Simple slope analysis between profession and susceptibility interaction revealed that, only for nursing students, susceptibility is a positive predictor of intention of getting vaccinated (b=0.14,se=0.05,p=0.003, 95% CI [0.05, 0.22]).

The independent variables were divided into seven blocks: (1) profession (medical or nursing student); (2) sociodemographic; (3) health-related variables that were found to be significantly correlated with intention to receive a COVID-19 vaccine in the univariate analyses; (4) all HBM; (5) all TPB variables; (6) interaction between profession and gender; and (7) interaction between profession and susceptibility. Here, we considered all two-way interactions between the profession variable and between HBM and TPB variables. In addition, we considered all two-way interactions that were found to be significant in the two-way ANOVA tests.

## 4. Hierarchical Logistic Regression Process

In the first step alone, the profession variable was inserted as a predictor. In the second step ($\Delta R^{2}=0.01,\;\Delta F\left(3,623\right)=2.38,p=0.07),$ the significant sociodemographic variables (gender and socioeconomic status) were inserted as predictors. In the third step ($\Delta R^{2}=0.03,\;\Delta F\left(3,620\right)=7.63,p<0.001),$ the significant health-related variables (COVID-19 infection and previous vaccination against influenza) were inserted as predictors. In the fourth step ($\Delta R^{2}=0.50,\;\Delta F\left(6,614\right)=122.16,p<0.001),$ all HBM variables were inserted as predictors. In the fifth step ($\Delta R^{2}=0.08,\;\Delta F\left(4,610\right)=34.49,p<0.001),$ all TPB variables were inserted as predictors. In the sixth step, we inserted all two-way interactions between the profession variable and all of the HBM and TPB variables as well as the gender variable. However, to avoid over-complexity and possible multi-collinearity, we used the stepwise procedure from the sixth step onwards. Thus, in the sixth step ($\Delta R^{2}=0.003,\;\Delta F\left(1,609\right)=4.90,p=0.03),$ only the two-way interaction between profession and gender was inserted. Lastly, in the seventh step ($\Delta R^{2}=0.003,\;\Delta F\left(1,608\right)=4.62,p=0.03),$ only the two-way interaction between profession and susceptibility was inserted. All other two-way interactions were excluded from the model via the stepwise procedure. Importantly, as accepted, we centered all variables that define the interaction products and calculated the interactions between them prior to inserting them into the regression model [32].

### Concerns Regarding the COVID-19 Vaccine

The main concerns of the respondents regarding the COVID-19 vaccine are provided in Table 4. Nursing students had significantly higher concerns than did medical students (all *p* values < 0.01). Specifically, nursing students had significantly higher preference for natural immunity than did medical students (M = 3.63, SD = 1.64 vs. M = 2.34, SD = 1.42, *p* < 0.1). The most common concern was the safety and quality of the vaccine (M = 4.52, SD = 1.49).

## 5. Discussion

The present study examined acceptance rates and predictors of medical and nursing students’ intention to receive a future COVID-19 vaccine. HCWs, including students, play an important role in the efforts against COVID-19, and a better understanding of their attitudes towards COVID-19 vaccination is of high importance. To the best of our knowledge, this is the first study to compare medical and nursing students’ intentions and attitudes towards COVID-19 vaccination, and to use an integrative model combining the HBM and TPB approaches.

The overall intention to receive a COVID-19 vaccine found in the present study was very high. However, medical students expressed higher intention of getting vaccinated against COVID-19 than did nursing students (88.1% vs. 76.2%). These results are compatible with the findings of Dror et al., who showed that, with regard to professionals (i.e., not students), vaccine acceptance among doctors (78%) was significantly higher than among nurses (61%) [11].

We examined several predictors for the intention to receive a COVID-19 vaccine, which were not previously studied in the context of COVID-19 vaccine acceptance among medical and nursing students, such as: socioeconomic status, periphery region. We also examined the interaction between profession and gender and found that males had higher intentions of getting vaccinated than females, but only among nursing students. This finding is in line with previous studies in the context of seasonal influenza, which showed that males had higher intentions to get vaccinated than females among nurses [33,34]. Our findings highlight the importance of focusing on female nurses when developing a targeted approach aimed at increasing vaccination rates, especially considering the fact that, in our study, female nurses constitute more than 80% of that profession.

We also considered the use of risk perception models by considering an integrated approach involving both HBM and TPB, in addition to sociodemographic and health-related variables. The resulting model was able to explain 66% of the variance in the intention to receive a COVID-19 vaccine among medical and nursing students, which was considerably higher than models based solely on HBM or TPB. This finding is consistent with previous studies conducted in the context of influenza, which reported that combined models can predict vaccination intentions and actual vaccination rates to a much greater extent than can each model alone [20,22,27].

According to HBM, susceptibility, benefits, cues to action and barriers were found to be significant predictors of the intention to receive a COVID-19 vaccine. Perceived susceptibility was identified as an important factor influencing the intention of getting vaccinated against COVID-19 [29]. Moreover, we examined the interaction between profession and susceptibility, and found it to be a good predictor for the intention to receive a COVID-19 vaccine among nursing students, but not among medical students. This finding highlights the need to increase the awareness of nursing students to their higher risk of being exposed to COVID-19.

Several cues to action have been presented in the literature as internal or external triggers, which may signal intention of getting vaccinated. Our study shows that medical and nursing students were more motivated to get vaccinated if they were recommended to do so by their family, friends, colleagues, supervisor or GP. This finding highlights the importance of recommendations and encouragement from supervisors in healthcare organizations, as was also pointed out by previous studies in the context of influenza [17,26].

Regarding benefits, we found that those who intend to receive the vaccine see high perceived benefits in obtaining the COVID-19 vaccine in terms of protecting themselves and others. This is similar to previous studies, which showed that vaccine acceptance relies on a personal risk–benefit perception [11], as well as a means to prevent transmission to patients and reducing the spread of the disease in general [17,24]. Avoiding absence from work was found as another motivation of getting vaccinated. This is not surprising, as the salary of medical and nursing students is typically not high, and therefore each working day is perceived as a high benefit [26,27].

Regarding barriers, our results were similar to those of previous studies conducted among HCWs. Specifically, concerns regarding the safety aspect of vaccination and adverse side effects, expressed fear of needles and pain, or lack of time, were associated with lower intentions of getting vaccinated [17,28]. When asked about concerns regarding the vaccine, nursing students had a significantly higher level of concerns about safety and side effects than did medical students. Nursing students also significantly preferred natural immunity more than medical students. Previous studies mentioned that some HCWs do not want to be vaccinated in the first round and would prefer to wait and see if there are any side effects [11]. A possible explanation for these concerns is the lack of information regarding the effectiveness, safety and quality of the COVID-19 vaccine at the time of conducting this study. Nevertheless, this finding highlights the importance of providing frontline nursing and support staff with up-to-date information on the COVID-19 vaccine, given the high degree of trust placed in them by patients. Moreover, additional educational efforts such as explanatory campaigns should be invested in nursing schools, which should include information about efficacy, safety and side effects of the vaccine.

According to our study, perceived severity and general health motivation were not found to be significant predictors. This result is consistent with previous studies reporting that severity of the disease was a much less significant predictor [22,27,32].

According to TPB, attitude, self-efficacy and anticipated regret were found to be significant predictors of the intention to get a COVID-19 vaccine. This finding is consistent with previous studies in the context of influenza vaccination, showing that positive attitude toward the vaccine was a strong predictor of the intention to get vaccinated [19,24]. Since HCWs and students serve as a role model to patients, it is important that they maintain a positive attitude towards the vaccine, as this attitude is reflected onto the patients.

The current study was conducted in Israel before COVID-19 vaccines had become available. At this point in time, many developed countries, including Israel, are already after massive COVID-19 vaccination campaigns. Therefore, the findings of this study are likely to have higher significance in the contexts of developed countries and future pandemics.

Finally, our study also addressed a concern with the influenza vaccine at the time of the COVID-19 pandemic. In this study, past vaccinations against seasonal influenza were correlated with the decision to get vaccinated against COVID-19. This is in agreement with previous studies that examined the intention to get a COVID-19 vaccine among the general public [35,36].

### Study Limitations

This study has several limitations that should be recognized when interpreting the results reported here. First, there is the time of distribution of the questionnaire. Specifically, the study was conducted before the vaccine was available. At that point, information on vaccine efficiency and safety was not definite. It is possible that, were the questionnaires distributed in December 2020, the degree of reporting of intent to vaccinate would have been different as the vaccine became available. Second, this study relies on self-reported questionnaires rather than objective measurement of actual vaccination, which is subjective in manner and can lead to a bias. Third, although the questionnaire was anonymous, it is possible that respondents answered in a manner that would allow them to be viewed more favorably, especially due to their role in the healthcare system, and therefore a social-desirability bias might be present. Lastly, this study analyzed an opportunity sample. Although the questionnaire was distributed digitally through social media groups consisting of medical and nursing students (e.g., Facebook and WhatsApp groups of several academic departments in Israel), we are unable to assess how many individuals were exposed to the questionnaire and chose not to fill it out. Such information might be important, as respondents are likely to have a stronger view, either positively or negatively, towards vaccination.

## 6. Conclusions

We found that the intention of getting vaccinated among medical students was higher than among nursing students. Moreover, we found that, among nursing students, the intention of males to get vaccinated was higher than that of females. These findings highlight the importance of paying attention and establishing intervention plans to deal with the latter group of female nurses, who expressed considerably low vaccine acceptance.

The use of HBM and TPB models is important for health policy makers and healthcare providers and can help better guide intervention programs. Specifically, we found the following predictors for the intention to receive a COVID-19 vaccine: high perceived susceptibility, benefits, barriers and cues to action, attitude, self-efficacy and anticipated regret. In particular, we believe that emphasizing the benefits of vaccination, at the same time as decreasing barriers, might have a great impact on vaccination rates.

The most common concerns regarding the vaccine were safety and quality. For these reasons, we presume that promoting vaccination campaigns that address the safety of the vaccine will have greater success than discussing the severity of the disease.

## Figures and Tables

**Table 1:** Comparison of baseline characteristics between medical students and nursing students, using chi-squared tests (*n* = 628).

Characteristics		Medical Students (*n* = 321)		Nursing Students (*n* = 307)		Total Sample (*n* = 628)		Statistics Analysis
		*n*	%	*n*	%	*n*	%	
Gender	Male	160	49.8	50	16.3	210	33.4	$\chi^{2}\left(1\right)=79.39,p<0.001$
	Female	161	50.2	257	83.7	418	66.6	
Relationship	No partner	118	36.8	184	59.9	302	48.1	$\chi^{2}\left(1\right)=33.76,p<0.001$
	Partner	203	63.2	123	40.1	326	51.9	
Children	No children	247	76.9	247	80.5	494	78.7	$\chi^{2}\left(1\right)=1.15,p=0.28$
	Children	74	23.1	60	19.5	134	21.3	
Ethnicity	Jewish	301	93.8	263	86.5	564	90.2	$\chi^{2}\left(1\right)=9.33,p=0.002$
	Muslim	20	6.2	41	13.5	61	9.8	
Religiosity	Religious	47	14.6	94	30.6	141	22.5	$\chi^{2}\left(2\right)=24.05,p<0.001$
	Traditional	72	22.4	65	21.2	137	21.8	
	Secular	202	62.9	148	48.2	350	55.7	
Periphery	Periphery	19	5.91	21	6.8	40	6.4	$\chi^{2}\left(2\right)=16.24,p<0.001$
	Between	91	28.3	132	43	223	35.5	
	Center	211	65.7	154	50.2	365	58.1	
Socioeconomic	Low	92	28.7	88	28.7	180	28.7	$\chi^{2}\left(2\right)=15.47,p<0.001$
	Medium	115	35.8	150	48.9	265	42.2	
	High	114	35.5	69	22.5	183	29.1	
Chronic Illness	Yes	54	16.8	52	16.9	160	16.9	$\chi^{2}\left(1\right)=0.001,\;p=0.97$
	No	267	83.2	255	83.1	522	83.1	
Live with someone in risk	Yes	64	19.9	114	37.1	178	28.3	$\chi^{2}\left(2\right)=22.93,p<0.001$
	No	226	70.4	168	54.7	394	62.7	
	Not sure	31	9.7	25	8.1	56	8.9	
Smoke	Yes	38	11.8	36	11.7	74	11.8	$\chi^{2}\left(2\right)=1.46,p=0.48$
	Past smoker	32	10.0	40	13.0	72	11.5	
	Never	251	78.2	231	75.2	482	76.7	
Corona-Infected	Yes	11	3.4	12	3.9	23	3.7	$\chi^{2}\left(2\right)=1.86,p=0.39$
	No	281	87.5	276	89.9	557	88.7	
	Not sure	29	9.0	19	6.2	48	7.6	
Exposure to corona patients at work	Yes	117	36.4	76	24.8	193	30.7	$\chi^{2}\left(1\right)=10.08,p=0.002$
	No	204	63.6	231	75.2	435	69.3	
Flu vaccine	Yes	262	81.6	146	47.6	408	65.0	$\chi^{2}\left(1\right)=79.99,p<0.001$
	No	59	18.4	161	52.4	220	35.0	
Flu illness	Yes	27	8.4	36	11.7	63	10.0	$\chi^{2}\left(1\right)=1.91,p=0.17$
	No	294	91.6	271	88.3	565	90.0	

**Table 2:** The main significant effects and interactions obtained in the Two-Way Analyses of Variance (ANOVA) among the sociodemographic and health-related variables.

Variable		M (SD)	*F*	*p*	$\mathrm{Partial}\;\eta^{2}$
Profession	Medical students	5.15 (1.37)	6.25	0.01	0.01
	Nursing students	4.56 (1.69)			
Gender	Male	5.08 (1.42)	3.04	0.08	0.01
	Female	4.75 (1.62)			
ProfessionxGender ^a^	Medical students. Male	5.07 (1.42)	8.13	0.01	0.01
	Medical students. Female	5.23 (1.33)			
	Nursing students. Male	5.12 (1.47)			
	Nursing students. Female	4.45 (1.71)			
SES	Low	5.05 (1.52)	3.12	0.045	0.01
	Medium	4.65 (1.67)			
	High	4.99 (1.40)			
COVID-19 infection	No	4.88 (1.54)	4.01	0.02	0.01
	Yes	3.96 (2.03)			
Previous flu vaccination	No	4.42 (1.70)	12.10	0.001	0.02
	Yes	5.10 (1.43)			

^a^ Represents the interaction effect.

**Table 3:** Coefficients of the final (seventh) step of the hierarchical regression.

Regression Blocks	Variable	b (se)	β	*p*	95% CI
	Constant	4.92 (0.08)		<0.001	[4.76, 5.08]
1	Profession ^a^	0.001 (0.09)	−0.001	0.99	[−0.18, 0.18]
2	Gender ^b^	−0.08 (0.09)	−0.02	0.37	[−0.26, 0.10]
	Low SES ^c^	0.12 (0.09)	0.08	0.20	[−0.06, 0.30]
	High SES ^c^	0.01 (0.09)	0.01	0.93	[−0.17, 0.19]
3	Had COVID-19 infection ^d^	−0.30 (0.20)	−0.19	0.14	[−0.69, 0.10]
	Doesn’t know if had COVID-19 infection ^d^	0.11 (0.14)	0.07	0.42	[−0.16, 0.39]
	Previous flu vaccination	−0.05 (0.09)	−0.02	0.53	[−0.22, 0.12]
4	Susceptibility	0.07 (0.03)	0.06	0.04	[0.002, 0.13]
	Severity	0.04 (0.05)	0.02	0.38	[−0.05, 0.14]
	Benefits	0.38 (0.05)	0.26	<0.001	[0.27, 0.48]
	Barriers	−0.23 (0.05)	−0.15	<0.001	[−0.32, −0.14]
	Motivation Health	−0.04 (0.04)	−0.03	0.24	[−0.12, 0.03]
	Cues to action	0.09 (0.03)	0.07	0.01	[0.02, 0.15]
5	Attitude	0.22 (0.04)	0.21	<0.001	[0.14, 0.30]
	Subjective Norms	0.07 (0.04)	0.06	0.10	[−0.01, 0.16]
	Self-efficacy	0.21 (0.05)	0.13	<0.001	[0.12, 0.30]
	Anticipated regret	0.16 (0.03)	0.17	<0.001	[0.10, 0.23]
6	Profession × Gender ^e^	0.41 (0.18)	0.06	0.02	[0.06, 0.76]
7	Profession × Susceptibility ^e^	−0.13 (0.06)	−0.05	0.03	[−0.25, −0.01]

^a^ Profession (0 = Nursing students, 1 = Medical students). ^b^ Gender (0 = Male, 1 = Female).^c^ SES (The reference group is “Medium SES”). ^d^ COVID-19 infection (The reference group is “No infection”). ^e^ Represents the interaction effects that were found to be significant in the stepwise regression analysis.

**Table 4:** COVID-19 vaccine concerns among medical and nursing students (*n* = 628).

Type of Concern	Medical Students (*n* = 321)		Nursing Students (*n* = 307)		Total Sample (*n* = 628)		*t* Value
	M	SD	M	SD	M	SD	
Temporary solution	3.79	1.29	4.22	1.22	4.00	1.27	4.27 **
Safety and quality	4.36	1.54	4.68	1.42	4.52	1.49	2.65 **
Not tried on others	4.17	1.59	4.64	1.43	4.40	1.53	3.88 **
Low efficiency	3.93	1.42	4.29	1.40	4.11	1.43	3.12 **
Natural immunity is preferable	2.34	1.42	3.63	1.64	2.97	1.65	10.54 **

** *p* < 0.01.

## Data Availability

The datasets generated during the current study are not publicly available but are available from the corresponding author on reasonable request.

## Supplementary Materials

The following are available online at https://www.mdpi.com/article/10.3390/vaccines9070783/s1, Table S1: HBM and TPB measures: comparison between medical and nursing students (*n* = 628).

## Abbreviations

CDC—Center for Disease Control and prevention; EUA—Emergency Use Authorization; FDA—U.S. Food and Drug Administration; HBM—Health Belief Model; HCWs—Healthcare Workers; PBC—Perceived Behavioral Control; TPB—Theory of Planned Behavior.
