# Supplementary material for: Integrating Health Behavior Theories to Predict COVID-19 Vaccine Acceptance: Differences between Medical Students and Nursing Students
Source: Vaccines (Basel). 2021 Jul 13;9(7):783. doi: 10.3390/vaccines9070783 (PMC8310115; doi:10.3390/vaccines9070783)
Supplement: Supplementary file 1 [file vaccines-09-00783-s001.zip › vaccines-1266238-SI.pdf]

Table S1. HBM and TPB measures: comparison between medical and nursing students (*N*=628). For all variables, except for general health motivation, cues to action and self-efficacy, medical students presented a significantly higher mean value, compared to nursing students.

Covariates		Medical students (*N*=321)		Nursing students (*N*=307)		Total sample (*N*=628)		*t* value
		M	*SD*	*M*	*SD*	*M*	*SD*	
Health Belief Model	Susceptibility	4.22	1.17	3.53	1.25	3.88	1.27	7.07**
	Severity	4.63	0.86	4.41	0.94	4.52	0.91	2.99**
	Benefits	5.18	0.90	4.74	1.22	4.97	1.09	5.15**
	Barriers	2.65	0.95	3.23	0.97	2.94	1.00	-7.53**
	Health Motivation	5.23	1.02	5.16	0.98	5.20	1.00	.80
	Cues to action	3.73	1.26	3.79	1.32	3.76	1.29	-.53
Theory of Planned Behaviour	Attitude	4.79	1.32	4.47	1.53	4.64	1.44	2.82**
	Subjective norms	5.18	1.03	4.55	1.35	4.88	1.24	6.55**
	Self-efficacy	4.48	0.95	4.34	0.97	4.42	0.97	1.78
	Anticipated regret	4.48	0.95	4.34	0.97	4.10	1.60	3.79**

**p<.01.
